# Two edges of the screen: Unpacking positive and negative associations between phone use in everyday contexts and subjective well-being

**DOI:** 10.1371/journal.pone.0284104

**Published:** 2023-04-26

**Authors:** Teodora Sandra Buda, Mohammed Khwaja, Roger Garriga, Aleksandar Matic

**Affiliations:** Research and Development, Koa Health, Barcelona, Spain; University of Otago, NEW ZEALAND

## Abstract

A plethora of past studies have highlighted a negative association between phone use and well-being. Recent studies claimed that there is a lack of strong evidence on the deleterious effects of smartphones on our health, and that previous systematic reviews overestimated the negative link between phone use and well-being. In a three-week long in-the-wild study with 352 participants, we captured 15,607 instances of smartphone use in tandem with rich contextual information (activity, location, company) as well as self-reported well-being measures. We conducted an additional study to gather users’ perception of the impact of phone use on their well-being in different daily contexts. Our findings show that context and personal characteristics greatly impact the association between screen time and subjective well-being. This study highlights the complexity of the relationship between phone use and well-being and it deepens our understanding of this problem.

## 1 Introduction

Smartphones are the most ubiquitous device in human history and the phenomena related to its use are unsurprisingly discussed across a wide range of scientific domains. One particular theme that fuelled an ongoing debate is the relationship between smartphone use and our well-being. For several years, the predominant view was almost unipolar—studies highlighted the undesirable impact of phone use, including social isolation [1], depression [2], stress [3]. However, recent criticism of previous studies brought an alternative view highlighting a lack of solid evidence for the previous claims around the negative impact of smartphone use [4], that moderate use of digital technology is not intrinsically harmful and may be advantageous in a connected world [5], and others report even benefits with greater screen time [6, 7]. Nevertheless, there is a general consensus that more research is needed on this topic that has a specific importance across multiple domains—from psychology and public health to human computer interaction.

To deepen our understanding in this area, the importance of solving the methodological issues rooted in self-reported screen time measurements has been demonstrated in multiple records [8–10]. Self-reports generally suffer from subjectivity, memory dependence and recall bias, and specifically when it comes to the phone use it has been shown that the greater the phone use, the greater the under-reporting of the screen time [8]. Automatically (and objectively) capturing the phone use brought more solid findings in this domain. Moreover, scientists explored thoroughly what is under the surface of the findings that previously suggested a negative link between the total (i.e., accumulated) screen time and well-being. In this regard, several studies focused both on what the users are doing in the virtual world (i.e., which specific apps such as music, games, productivity apps, etc) [7], a few studies focused on what the users were doing in the physical world while using the phone [11], and a recent study explored both aspects [12]. How the physical context (i.e., what the users where doing concurrently with the phone use, where, and with whom) as well as interpersonal differences (in terms of personal characteristics, typical daily routines, and habitual phone use patterns) impact the relationship between the phone use and well-being remain unanswered.

Inspired by prior work, in this paper we unravel the unexplored detail around the ways that mobile phone users’ personalities and the real-world activities and context associated with phone use contribute to their subjective perception of well-being. We captured screen time from smartphone sensors, along with rich contextual information (activities, location and company) through Ecological Momentary Assessments delivered five times a day over a period of three weeks from 352 participants. We further extend and thoroughly analyse the associations between screen time and well-being by considering various additional factors, as well as the interplay between them. These include contextual information such as detailed descriptions of activities, surrounding people (also referred to as *company*), location, and time of the day, as well as personal characteristics (personality, gender, age).

Understanding the relationship between the use of smartphones and subjective well-being is of a particular importance to Human Computer Interaction (HCI) research. Designing interfaces to enhance user’s emotional well-being in a digital service lead to its increased use, therefore the designers of digital services have been lately focusing more on making services enjoyable rather than solely optimising for a usability [13]. A recent framework for designing digital experiences [13] highlighted “designing for well-being” as one of the three key designing principles (in addition to motivation and engagement). The lack of understanding of the underpinning mechanisms in this relationship leaves the HCI researchers ill-equipped to leverage this knowledge for designing smartphone services that better promote well-being. Our results brings the attention for considering different physical contexts and interpersonal differences when designing services that aim to promote well-being and thus a higher engagement.

## 2 Related work

Subjective well-being (SWB) refers to how people evaluate and experience their lives [14]. The literature also distinguishes two important facets of SWB, eudaemonia and hedonia [15, 16], colloquially known as feel-purpose vs. feel-good. Hedonistic well-being is related to positive and negative experiences such as happiness or sadness, whereas eudemonic well-being represents a sense of purpose and meaning in life. Measuring eudaemonia and hedonia associated to activities in everyday life is based on asking people to report how happy vs how worthwhile they felt during an activity [15]. In our study, participants were asked to report both the happiness and worthwhileness in each self-report. We believe that distinction between the happiness and worthwhileness is relevant when studying well-being in the context of smartphone use given a multitude of smartphone applications that may have a different impact on these two facets. Although there is no clear-cut categorisation of different applications along the two well-being facets, one may argue that being involved in productivity, learning, or work related activities may impact more “feel-purpose” whereas music, videos, games and similar may impact more “feel-good” constructs. Moreover, user’s ratings of the two constructs may be very different even with the same kind of activities e.g. using social media to talk to a colleague or to a friend can feel differently in terms of productivity / worthwhileness vs pleasure / happiness. Therefore, we considered both dimensions of subjective well-being relevant for this study, tapping into the associations between SWB and smartphone use.

Smartphones are tightly embedded in our everyday life, and an ample interest in the effects of their use both by the scientific communities and the media does not come as a surprise. Previous work highlighted a myriad of negative effects of smartphone usage both in the context of a typical as well as of atypical (usually referred to as excessive or problematic) phone use. Undesirable impact was reported in the relationship with well-being [17], cognitive development [18], and mental health [19–22], spanning to high correlations even with symptoms of mental health disorders including depression [2] and anxiety [23]. The negative effects have been confirmed also in longitudinal studies suggesting that increases in recreational screen time precede a lower psychological well-being [24–30]; refraining from using social media for even just one week was associated with an increase well-being [31], and decrease in anxiety and depression [32]. Moreover, several studies have shown that the presence of smartphones can negatively impact enjoyment during social interactions [33, 34]. Nevertheless, a vast number of existing studies rely on *self-reported* smartphone usage metrics, which was one of the core methodological issues that called into question the previous findings and even opened a new research theme on exploring misreports of the smartphone use.

In a study focusing on smartphone addiction [8], the authors demonstrated that self-reported duration of smartphone use is significant lower than the automatically quantified smartphone use captured via an application that logs every phone usage. The degree of underestimation was positively correlated with actual smartphone use (i.e., the more the participants used their smartphone, the greater the extent of underestimating its usage duration). A potential reason behind the underestimation of the self-reports is the negative image associated to the overuse of smartphones [35], as suggested after studying this phenomenon from various disciplines (sociology [36], psychology [19], and medicine [37] as well as propagated in social media [38]). Either consciously or subconsciously, smartphone users tend to conceal an image of themselves overusing or in extreme cases being addicted to their smartphone. Along this line, a few studies specifically analysed users’ perception of smartphone usage [9, 10] and report over/underestimation of self-declared usage as compared to actual usage. These findings challenge previous studies relying on self-reported measures of smartphone usage and suggest that their findings need to be taken with reservation and the relationship between the phone use and mental well-being should be explored further by relying on automatic quantification.

This prompted a great deal of studies to delve deeper into the links between well-being and *passively* measured screen time. Mehrotra et al. [12] examined the relationship between smartphone interaction and users’ emotional state. Similar to our study, they collected objective measures of smartphone use data (such as app use, notifications, interactions with the phone) as well as self-reported emotional states through experiential sampling. In addition, they included weather information, activity extracted from Google Activity Recognition API [39] (i.e. classifying the user’s activity into walking, bicycling, commuting on vehicle or still), and home and work locations. Among other findings, they reveal that people become more attentive and respond faster to notifications in stressful situations whereas an increase in users’ activeness level reduces the usage of music apps.

In a similar line, Sarsenbayeva et al. [40] explored the relationship between user’s emotions and smartphone use based on objective measures of smartphone use data, collected together with emotional states modelled from user’s facial expressions (passively captured using the smartphone’s front-facing camera). The study revealed that phone use typically drives certain emotions rather than the other way around, although they also identified cases in which application use drives emotions. The authors argued that the causal relationship between emotional state and smartphone use varies among people as emotions and behaviours are intrinsically personal. Moreover, the qualitative survey that they administered revealed various emotions when using the same app which depended on the content.

Recently, scientists argued that there is a lack of strong evidence on the detrimental effects of smartphones use on our mental health [41, 42] and that previous studies and systematic reviews overestimated the negative associations [4]. In this regard, Orben et al. [43] found that digital-technology use has a small negative association with adolescent well-being. In particular, the strength of the association between screen time and well-being was similar to neutral factors and the authors compared it to wearing glasses or regularly eating potatoes. Similar studies found null effects or even benefits with greater screen time [6, 7, 44–46]. Moreover, in [5], Przybylski et al. reveal that the moderate use of digital technology is not intrinsically harmful and may be advantageous in a connected world. We built on and expanded the previous work by decoupling the relationship between phone use and subjective well-being along two different factors, namely considering: contextual information (activity, location, company, and time of the day), personal characteristics (personality, age, gender).

## 3 Methods

To thoroughly investigate the associations between smartphone usage in various daily contexts and users’ self-reported subjective well-being, we applied a mixed-methods approach by conducting:

1. A quantitative study in which we passively quantified smartphone usage which was coupled with users’ self-reports (Section 4) administered as Ecological Momentary Assessments (EMAs) [47], and
2. A qualitative survey, in which we probed the perceived subjective well-being associated to smartphone usage in different daily contexts (Section 6).

The goal of the quantitative study was to understand associations between objectively measured screen time and self-reported well-being measures, with respect to contextual information, personal characteristics, and individuals’ patterns of using the phone.

To complement the quantitative analysis, we administered an extensive survey with an objective to understand which contexts users associate with more positive or more negative emotional responses. The survey was designed considering the quantitative study—we selected the activities, companies and locations that were most frequently reported or that were linked to the polarised emotional responses.

Therefore, we delved into the associations between phone use and subjective well-being both from a subjective self-reflection standpoint as well as from the statistical analysis using longitudinal data captured in-situ. In addition, we also analysed the users’ declarative statements to acquire a descriptive layer of the same phenomenon in Section 6.3.

## 4 Quantitative study

### 4.1 Data collection

**Fig 1:**
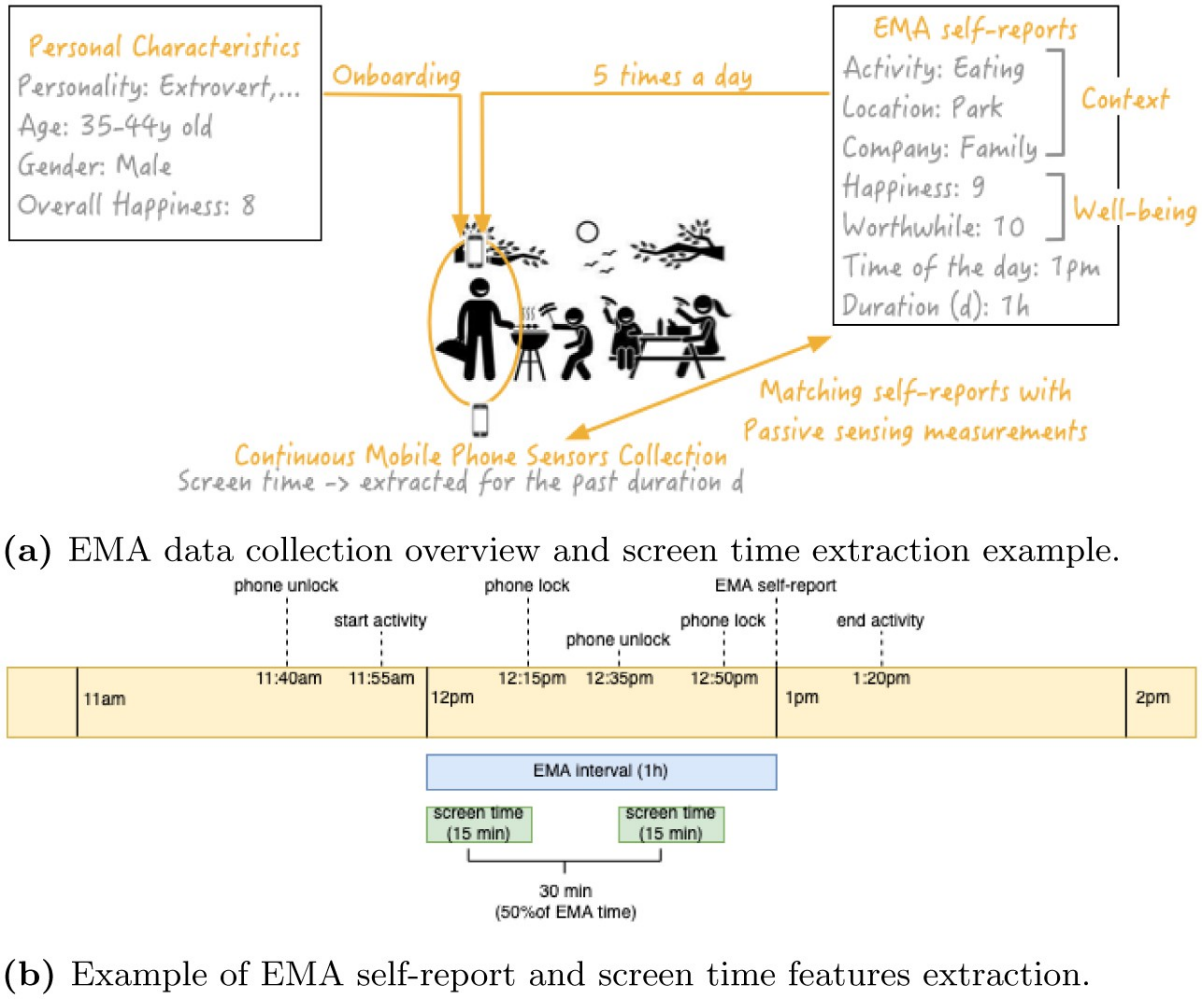
Data collection overview. (a) EMA data collection overview and screen time extraction example. (b) Example of EMA self-report and screen time features extraction.

The data for this study was obtained using a custom-built Android App, designed to collect passively phone usage events from smartphone sensors and well-being from self-reported questionnaires. Phone usage events included the timestamps when the phone was unlocked, and when the screen was turned on and off. Data was sampled every time one of these events occurred. Using Ecological Momentary Assessments (EMAs), the app prompted users to rate two dimensions of subjective well-being—happiness and worthwhileness (i.e., hedonic and eudemonic components). The momentary subjective well-being was rated on a 1 to 10 scale (1 being the lowest) at five instances each day during the study. Notifications were sent at a random time within five time windows: 8:00 AM–10:00 AM, 10:30 AM–12:30PM, 13:00 PM–3:00 PM, 3:30 PM–5:30 PM, 6:00 PM–8:00 PM. EMA notifications were not sent during night time in order not to interfere with sleep. If a user did not attend an EMA prompt, a reminder was sent after 15 minutes. A 30 min gap between each time slot prevented overlaps in subsequent EMA reports, and to provide users with enough time to answer the questions. Fig 1a shows an overview of the data collection categories in an illustrative scenario. In addition to well-being reports, EMAs included reports of the momentary context (activities, location and company). To obtain their context, we asked users: (a) what they were doing, (b) where they were, and (c) who they were with. Users could select multiple items from a list containing common activities, locations and companies. The report had an option to report activity, location and company items in a free-text format in case the items already in the list did not suffice. The language used for EMA prompts was English for English speaking countries and Spanish for Spanish speaking countries.

Participants were asked to use the app for a period of 3 weeks. In compliance with the EU’s GDPR, participants were presented with a consent form describing details of the data collection and purpose of the study upon installing the app. Thereafter, participants completed an onboarding process in which we captured basic demographic questions (gender, age, employment status, etc.) and the Big Five personality traits using the 50 item-International Personality Item Pool (IPIP) questionnaire [48]. No personal identifying information, such as name, email address etc., were collected during this step.

### 4.2 Data processing and features extraction

#### 4.2.1 Contextual information extraction

As we outlined in Section 4.1, the user may report multiple activities, locations and company in each EMA. In all three context categories, the majority of EMAs reported only one instance. In particular 86.3% of the EMAs reported a single activity, 97.4% reported only one location and 92.2% a single company. For our analysis, we treated each of the activities reported in the EMA as different data instances and kept the same start and end of intervals. For simplicity, we assigned the first reported location and company to all the data instances corresponding to the EMA. There were a total of 53,244 EMAs collected, which were used to generate 62,221 data instances.

#### 4.2.2 Screen time features extraction

For the purpose of our study, we extracted the *total screen time duration* and *percentage screen time duration* in each of the data instances corresponding to EMA reports. As first step, phone usage events were utilized to extract sessions of phone usage. We labelled the timestamp of each unlock event as the start of the session and the timestamp of the subsequent screen-off event as the end of this phone use session. Screen time duration of each session was directly computed as the number of minutes between the start and the end of the session. A total of 1,493,553 phone usage events were collected, which were transformed into 431,044 sessions.

Similarly, we processed the data collected through EMAs to build the time interval that the reported contextual information belonged to. For instance, considering the exemplary EMA report from Fig 1a, the time interval would be from 12:00 PM to 1:00 PM as the recorded EMA timestamp (1pm) and the duration reported (1h). Due to the momentary nature of EMAs, the exact moment at which the activity, company or location changed was unknown (due to the fact that EMAs, by design, frequently arrive in the middle of an activity). For this reason, we only considered the time period until the user finished the EMA and we treated the logged timestamp as the end of the interval. Therefore the logged timestamp is considered the end of the activity and other reported contextual information. The start of the interval was computed by subtracting the reported duration from the EMAs timestamp.

Next we proceed with extracting the screen time features per EMA report. Let us consider a person A commuting for an average of 120 minutes to get to work with a moderate total phone usage duration during this period of 60 minutes, equivalent to 50% of percentage duration of screen time, against a person B commuting for an average of 10 minutes to get to work with a moderate total phone usage duration during this period of 5 minutes, equivalent to 50% of percentage duration of screen time. There is a clear difference between the two examples in terms of total duration of screen time, while the difference disappears when considering the percentage duration of screen time. This highlights the importance to consider both metrics. We computed the total screen time duration per EMA report. For each data instance, the total screen time duration was obtained by finding all the sessions of phone usage that overlapped with the EMA’s interval and by aggregating the amount of time they overlapped with that interval. This resulted in one of our target variables: the *total screen time duration*. The total screen time gives the absolute effect of the number of minutes spent using the phone while each specific context (e.g., activity, location, companion, time of day) is ongoing; with it, we aimed to discover the effect of spending more/less minutes using the phone while in the context. Secondly, we compute the proportion of time the user spent on the phone during an EMA interval by dividing the total screen time duration with the EMA reported duration. This resulted in the second target variable for our analysis: the *percentage screen time duration*. The percentage screen time duration gives the relative effect of using the phone while in each context, which is important in this study as well because the self reports vary in length; with it, we aimed to discover the effect of spending a larger/smaller part of the time in the context using the phone. For instance, considering the exemplary timeline from Fig 1b, the user unlocked the phone at 11:40, locked it at 12:15, unlocked it at 12:35 and locked it again at 12:50. However, since the self report was filled at 1pm and the activity duration was reported to be 1h, the time interval used to compute the screen time features is between 12pm and 1pm, which results in a *total screen time duration* of 30 minutes and a *percentage screen time duration* of 50%.

#### 4.2.3 Context and personal characteristics categorization

From the self-reported activities, companions and locations, we obtained the categories that were frequently reported to use as different contexts in our analyses. As users were allowed to input their own categories through a free text entry, we disregarded categories that have less than 100 entries in to analyse the statistical significance between groups. Moreover, we included in the categories of personal characteristics available from the onboarding questionnaire, such as gender, age and personality. We expanded the contexts reported with the time-based categories in order to explore the association between phone use and well-being at certain time windows during the day, as well as during holidays. We categorised the time when the EMA was completed into: morning (from 6AM to 12PM), afternoon (from 12PM to 6PM), evening (from 6PM to 12AM), and the day as a holiday if it was a weekend or public holiday as well as if the user reported as being on holidays. Since we did not send notifications during sleeping hours, we only had 7 EMA reports filled at night (despite of not sending the reminders), hence this time interval was excluded from our analysis. With respect to personality, we utilized the answers from the Big Five questionnaire [48] and included all the five traits for our analysis (i.e., Extroversion, Agreeableness, Conscientiousness, Neuroticism and Intellect). The traits were normally distributed (we tested normality with the Shapiro-Wilk normality test [49] to verify if the distributions were Gaussian). Further, we used the median value to split participants into two groups, e.g., into High Agreeableness versus Low Agreeableness. This methodology has been previously applied in the literature [50, 51] to analyse different clusters of individuals. The exhaustive categories considered for our analysis are shown in Table 1.

**Table 1:** Contextual and personal characteristics categories.

Group	Categories
Activity	Browsing Internet, Commuting, Conversation, Eating, Emails, Exercise, Listening to Music, Reading, Shopping*, Social Media, Studying, Taking a Nap, Taking a Shower, Waiting, Watching TV**
Company	Alone, Colleagues, Family, Friends, Kids, Partner, Not Alone, Unknown People
Location	At friends’ house, At parents’ house, Home, Restaurant, Sport facility, Street/Outdoors, Supermarket, Work***
Time	Morning, Afternoon, Evening, Holiday
Age	18–25y old, 26–34y old, 35–44y old
Gender	Male, Female
Personality	Extroverts, Introverts, Neurotics, Emotionally Stable, High and Low {Agreeableness, Conscientiousness, Intellect}

* Instances corresponding to the Activity—Shopping category refer to both online and onsite shopping.

** Instances corresponding to the Activity—Working category were merged with Location—Work

***1. Location—Public transport was merged with Activity—Commuting & 2. Location—University was merged with Activity—Studying, since they refer to the same contextual category and to avoid unnecessary sparsity across categories

### 4.3 Participants and inclusion criteria

In total, the data from 921 users was collected for this study. The participants were recruited from five different countries (Chile, Colombia, Peru, Spain and the United Kingdom) by an external recruitment agency from February to August 2018. Participants were asked to use the application for a period of 3 weeks, and upon a successful completion of the study they were rewarded with a monetary compensation of EUR 40.

Table 2 shows the number of individuals and data instances after each step of the inclusion criteria process. For the analysis, we first excluded participants that did not provide any EMA self-reports (i.e., who solely completed the onboarding questionnaire) and participants whose logs did not contain any sensor or unlock events for computing screen time. Furthermore, we removed the incomplete EMA records that did not contain a reported well-being score. After applying the inclusion criteria, the final dataset used for our analysis included in total 352 participants and 15,607 EMA records, out of which 344 participants provided their gender (129 Females, 215 Males) and age. In particular, over a half of the participants were aged between 26–34 (N = 237), while others belonged to various age ranges, including 35–44 (N = 70), 18–25 (N = 33), 45–54 (N = 1), 55–64 (N = 2) and 65+ (N = 1).

**Table 2:** Breakdown of the inclusion criteria process.

Inclusion criteria step	# Users	# Instances
Original	921	62,221
With EMAs	873	62,221
With sensor events	479	33,610
EMAs with well-being	352	15,607

### 4.4 Methodology

#### 4.4.1 Correlation analysis

As the first step in the analysis, we performed a correlation analysis using Spearman’s rank correlation coefficient between the well-being scores provided by the users in the EMA reports and the corresponding (passively captured) screen time measurements depending on the main categories defined in Table 1.

#### 4.4.2 Multiple hypotheses correction

In order to control for the false discovery rate, we used the two-stage step-up method of Benjamini, Krieger and Yekutieli [52]. This technique is an adaptive method that controls the false discovery rate by incorporating an estimate of the number of true hypothesis based on the distribution of p-values. We applied this correction separately for the correlation p-values of Total duration and Percentage duration of screen time. We utilized a maximum threshold for the p-values computed of 0.05 for evaluating statistical significance for both before and after correction for multiple hypotheses (50 hypotheses evaluated, based on the categories specified in Table 1).

#### 4.4.3 Regression analysis

Third, in order to leverage the fact that the activity, location and company contexts are features that capture the state of a user, we train linear mixed effects regression models for analysing the relationship between different aspects of phone usage and momentary subjective well-being. Mixed linear regression models with fixed effects were used to analyse the relationship between different aspects of phone usage and momentary or experienced happiness reports as dependent variable in the following configurations: (1) screen time features only, (2) screen time features with activities and their interaction, (3) screen time features with activities and company features and their interaction, and finally (4) screen time features with activity, company and location and their interaction. For all models, we utilised the user identifier as group to leverage that we have multiple EMA reports for each user. When using all the categories from Table 1 and their interactions, the model failed to converge, as coefficients and statistical significance were missing for less popular categories. To address this issue, we removed categories with less than 500 instances. In particular, the following categories were included in the regression analysis: (a) Activity: ‘Social Media’, ‘Eating’, ‘Watching TV’, ‘Browsing Internet’, ‘Conversation’, ‘Waiting, ‘Listening to Music’, ‘Studying’, ‘Commuting’, ‘Taking a nap’ (b) Company: ‘Alone’, ‘Kids’, ‘Partner’, ‘Family’, ‘Friends’, ‘Colleagues’ (c) Location: ‘Home’, ‘Street/Outdoors’, ‘Work’. The variables included in the final model were the ‘Total phone usage (min)’, ‘Total phone look (min)’, all the categorical variables from (a), (b) and (c) and all interactions between ‘Total phone usage (min)’ with the categorical variables from (a), (b) and (c).

## 5 Quantitative study: Results

### 5.1 General descriptive statistics

**Fig 2:**
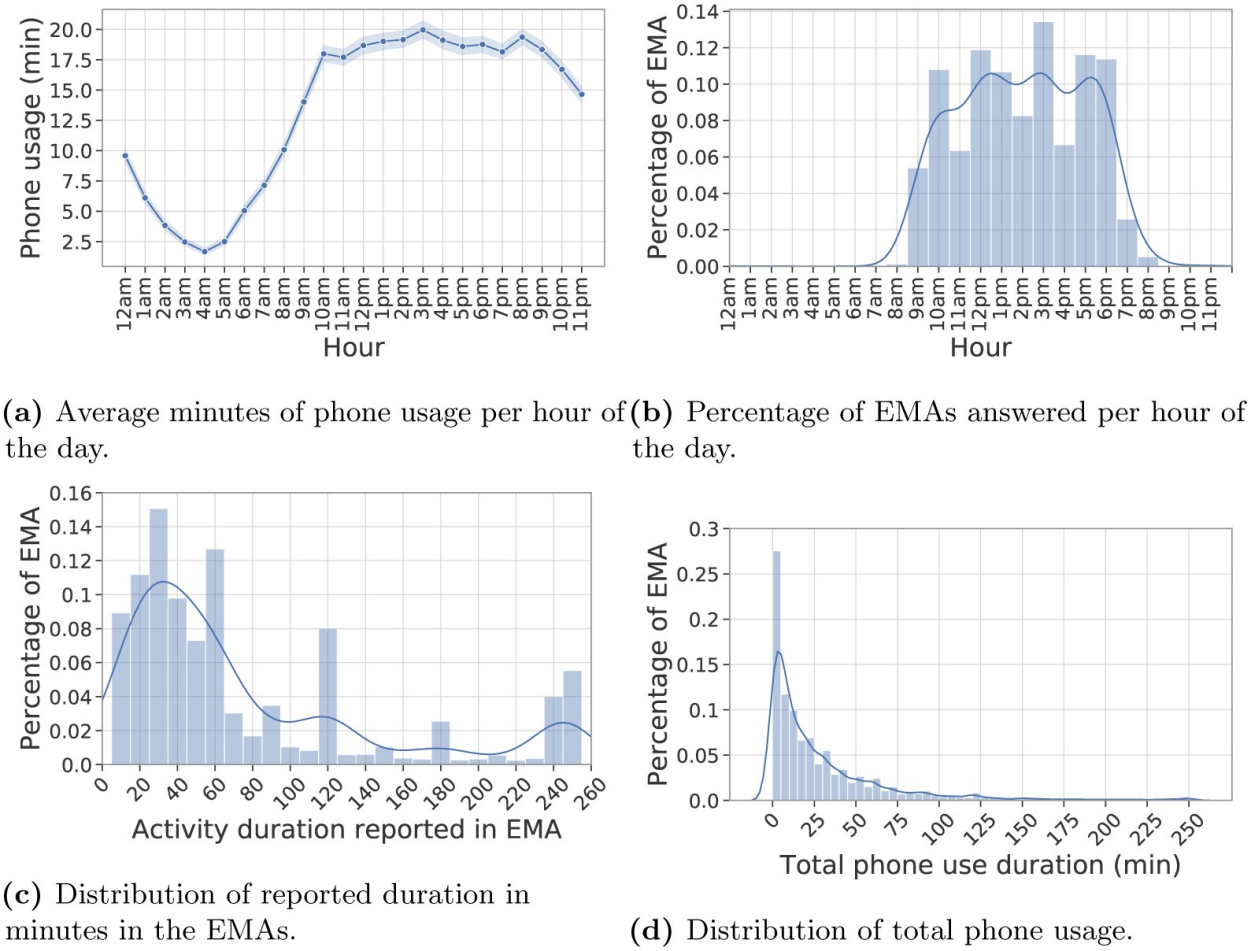
Patterns of phone usage and EMA reports. (a) Average minutes of phone usage per hour of the day. (b) Percentage of EMAs answered per hour of the day. (c) Distribution of reported duration in minutes in the EMAs. (d) Distribution of total phone usage.

The data collected included 53,244 EMAs and 1,493,553 phone usage events from a total of 921 participants. We analysed the phone usage patterns and EMA responses per hour of the day and visualized it in Fig 2. As shown in Fig 2a, our participants start using the phone between 6am and 7AM and their phone usage grows steadily until 10AM. The average number of minutes using the phone remains stable between 17.5 and 20 minutes per hour until 8PM and declines from that time until its minimum at 4AM. These patterns are in line with previous findings [53–55]. Fig 2b shows a visualization on the percentage of EMA’s answered per hour of the day. Participants received notifications between 8AM and 8PM, as described in Section 4.1. The EMAs were answered quite evenly between 11AM and 8PM, with a peak at 5PM and a decline after 8PM similarly to the phone usage.

Further, we analysed the duration of activities reported on EMA and the phone use while the activity was realised. As shown in Fig 2c, a large portion of reported activities lasted for 60 minutes or less, accounting for 65% of EMAs. Additionally, it shows a peak every 30 minutes that is more pronounced at each natural hour, and for a portion of activities (almost 6%), the users reported that the activities lasted 250 minutes, which was the maximum allowed to report. Fig 2d shows the distribution of total minutes of phone usage calculated based on the corresponding reported EMA intervals. The participants used their phone a median of 15 minutes while performing the reported activity and for a period of less than 30 minutes in almost 70% of the cases.

**Fig 3:**
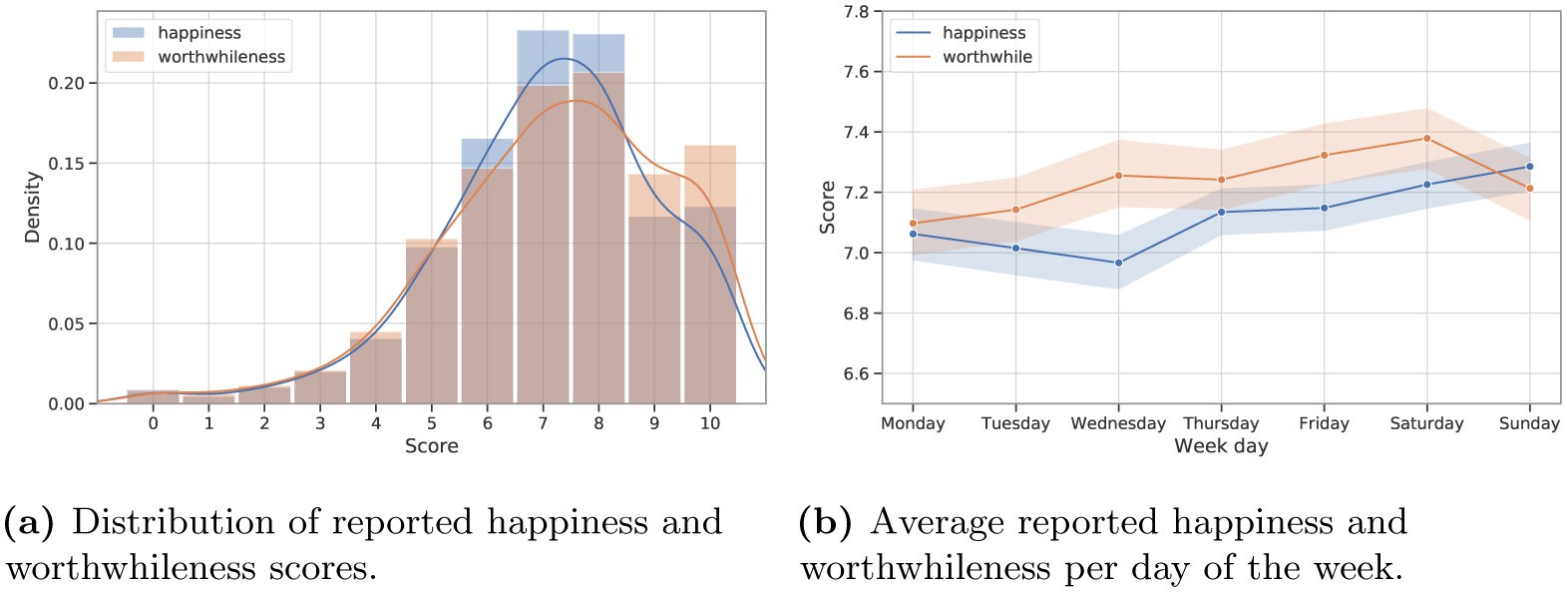
Reported subjective well-being measures. a) Distribution of reported happiness and worthwhileness scores. (b) Average reported happiness and worthwhileness per day of the week.

Finally, we analysed the subjective well-being of our participants, measured through reported happiness and worthwhileness scores, in the different contexts and their evolution during the week. Both happiness and worthwhileness measures have a similar distribution as shown in Fig 3a, and are highly correlated with a Spearman correlation value of 0.76 (p-value <0.001). The distribution is skewed towards larger values with a median value of 7 for both measures. During weekends, the reported happiness is significantly higher than during the rest of the week (p-value <0.01) and there is a negative trend on the happiness score from Monday to Wednesday, Wednesday being the day with the lowest score. These insights are inline with previous studies that show similar trends [56, 57]. Worthwhileness scores show a positive trend during the week until Saturday and are generally higher than happiness scores, except on Sundays, when the worthwhileness scores decrease. Previous studies have identified that context plays a role in momentary subjective well-being [58, 59]. In our study, we also observed that participants reported higher subjective well-being scores on certain context categories with respect to others. In particular, users reported higher levels of happiness and worthwhilness while doing activities such as Exercise, Taking a shower and Having a conversation, and lower levels while Waiting, Browsing Internet/Social Media/Emails and Commuting. Sport facilities and Street/Outdoors were locations where participants reported higher subjective well-being, while they reported lower scores in Work, Home and Friends’ house. When participants were in company of their Kids or Friends their subjective well-being scores were higher and lower when Alone or with Unknown people. Please S1–S3 Figs for a complete breakdown of reported subjective well-being based on the context categories studied.

### 5.2 Associations of phone use with subjective well-being

#### 5.2.1 Correlation analysis

**Fig 4:**
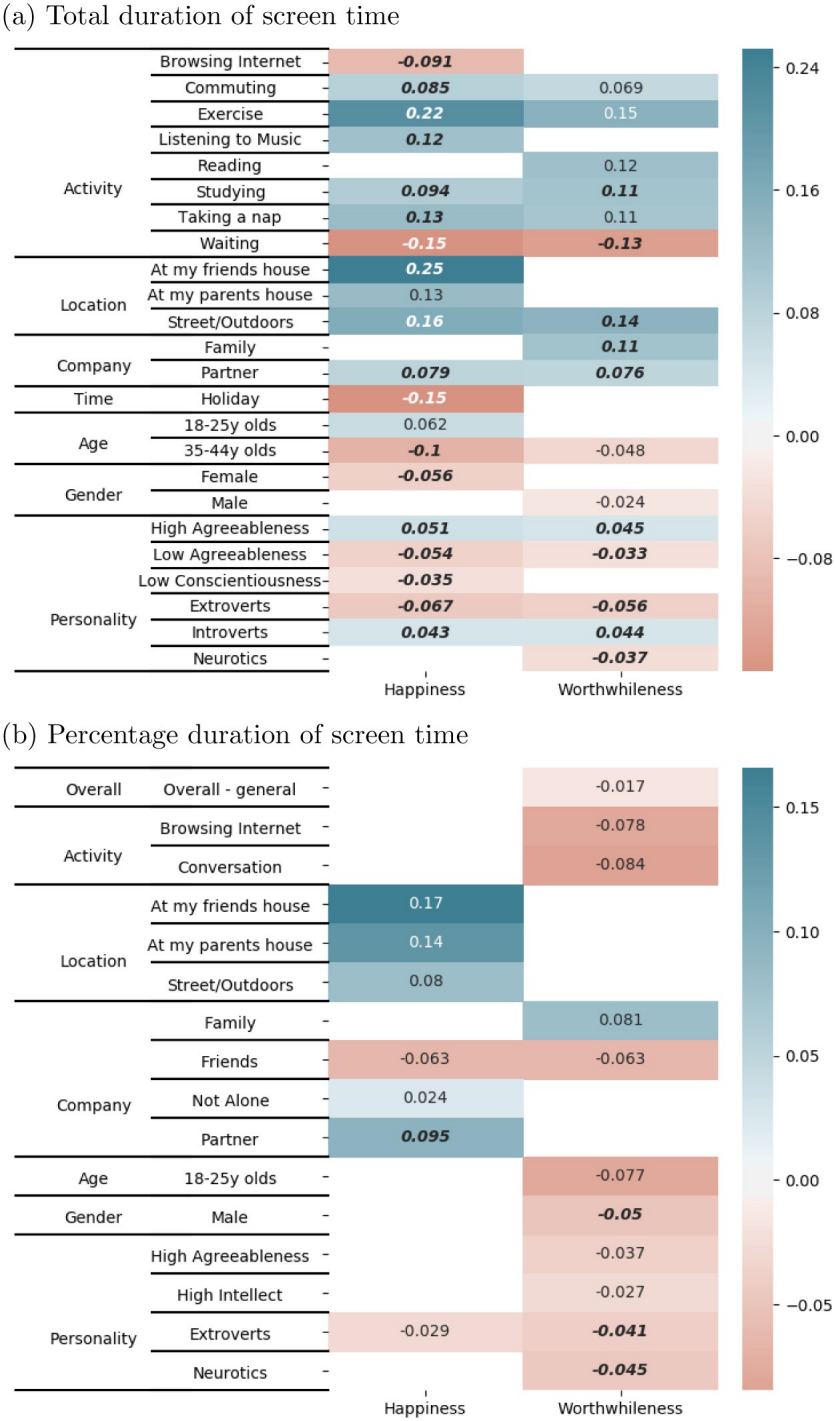
Significant Spearman correlation results of total duration of screen time (a) and percentage duration of screen time (b) with reported happiness and worthwhileness. Correlations that remain significant after multiple hypothesis correction are illustrated with bold and italic font.

Fig 4 shows the significant Spearman correlation coefficients that are computed to assess the relationship between well-being self-reports and total duration of screen time and percentage duration of screen time. We discuss why we considered both metrics in Section 4.2.2. The figure is color-coded according to the valence of relationship and its effect size: darker colors indicate a stronger effect; blue indicates that a positive correlation between well-being and phone use, and orange indicates a negative correlation between well-being and phone use.

Overall, we observed a significant low negative correlation between phone use and worthwhileness, yet only before correction for multiple hypotheses. Adding context as a dimension revealed several siginificant positive associations between phone use and well-being, after correction for multiple hypotheses: (a) when performing certain activities such as studying, listening to music, exercising, commuting, taking a nap, (b) when being in the presence of family, or partner, (c) when being outdoors, or at their friends house, (d) for introverts, or individuals with high agreeableness. Moreover, we observe negative associations between phone use and well-being: (a) when browsing internet, waiting, (b) when being on holidays, (d) for 35–44y olds, (e) across both female and male genders, and (f) being an extrovert, or neurotic, or individuals with low agreeableness, or low conscientiousness.

We observe that context plays a significant role when evaluating the associations between screen time and well-being. Overall, we observe more positive associations rather than negative ones from correlations. Beyond activity, location, company, and time contexts, personality and age appear as well significant when considering the association between phone use and well-being.

#### 5.2.2 Regression analysis

The results are presented in Table 3. We included only the interactions that were significant between screen time and categories. The total duration of screen time was significant (*p* < 0.01) in the first model only, and its effect disappeared when contexts and interactions were added. These results further suggest the importance of contexts in the association between screen time and subjective well-being. Yet, due to the design of this particular study, we refrain from making conclusions related to the causal link between screen time and the subjective well-being.

**Table 3:** Regression coefficients, significance (*: p< = 0.05, **: p< = 0.01, ***: p< = 0.001) and clustered standard errors (in parentheses) for fixed effects models on experienced happiness for (1) basic model with screen time features only, (2) model with screen time and activities and their interaction, (3) model including screen time, activity, company and their interaction, and (4) model including screen time variables, all contexts (activity, company, location) and interactions.

DV: Experienced happiness	(1) Screen time	(2) Screen time with activities and interactions	(3) Screen time with activities, company and interactions	(4): Screen time with activities, company location and interactions
*Screen time*:				
*Total phone usage (min)*	-0.001 (0.000)***	-0.001 (0.001)	-0.004 (0.003)	-0.003 (0.003)
*Total phone look (min)*	-0.001 (0.001)	0.000 (0.001)	0.001 (0.001)	0.000 (0.001)
*Activity*:				
*Social Media*		0.037 (0.065)	0.009 (0.067)	0.001 (0.067)
*Eating*		0.317 (0.045)***	0.200 (0.048)***	0.185 (0.049)***
*Watching TV*		0.190 (0.054)***	0.086 (0.056)	0.079 (0.058)
*Browsing Internet*		0.104 (0.062)	0.071 (0.063)	0.060 (0.064)
*Conversation*		0.396 (0.061)***	0.127 (0.066)	0.125 (0.066)
*Waiting*		-0.180 (0.066)**	-0.290 (0.067)***	-0.307 (0.067)***
*Listening to Music*		0.165 (0.074)*	0.134 (0.075)	0.139 (0.075)
*Studying*		-0.104 (0.071)	-0.275 (0.073)***	-0.294 (0.075)***
*Commuting*		-0.099 (0.062)	-0.114 (0.063)	-0.203 (0.067)**
*Taking a nap*		0.325 (0.074)***	0.280 (0.075)***	0.271 (0.076)***
Activity * Screen time				
*(with interactions)*:		-0.008 (0.002)***	-0.007 (0.002)***	-0.008 (0.002)***
Waiting * Total Duration				
Eating * Total Duration		-0.003 (0.001)*	-0.004 (0.001)**	-0.004 (-0.001)*
Browsing Internet * Total Duration		-0.003 (0.001)*	-0.003 (0.001)*	-0.003 (0.001)*
*Company*:				
*Alone*			-0.347 (0.133)**	-0.323 (0.133)*
*Kids*			0.362 (0.154)*	0.365 (0.154)*
*Partner*			0.055 (0.139)	0.053 (0.139)
*Family*			-0.207 (0.143)	-0.176 (0.143)
*Friends*			0.307 (0.139)*	0.306 (0.138)*
*Colleagues*			-0.425 (0.138)**	-0.263 (0.142)
Company * Screen time				
*(with interactions)*:			0.009 (0.003)**	0.008 (0.003)**
Family * Total duration				
*Location*:				
*Home*				-0.066 (0.045)
*Street/Outdoors*				0.069 (0.071)
*Work*				-0.275 (0.069)*
Location * Screen time				
*(with interactions)*:				0.006 (0.002)***
Street/Outdoors * Total duration				
Number of observations	13981	13981	13652	13448
Number of individuals	350	350	350	326

The fixed effects models take into account the effect of each context and its interaction with the screen time, showing numerous significant correlations with the experienced happiness. The positive associations of fixed effects that were significant in all the models in which they were included are: the activities eating and taking a nap and being in the company of friends or kids. In contrast, the negative associations that were significant in all models are: waiting, being alone and being at work. There were some significant associations that disappeared as new context categories were added. For instance, watching TV, having a conversation or listening to music were positively associated at a significant level only when company and location were not considered. These activities often involve the company of other people, which might be the reason why these effects disappear when company categories are included. Also, the company of colleagues had a significantly negative association (*p* < 0.01), but when location was incorporated it disappeared—possibly because the association was related to being at work where people share space with colleagues most of the time.

**Fig 5:**
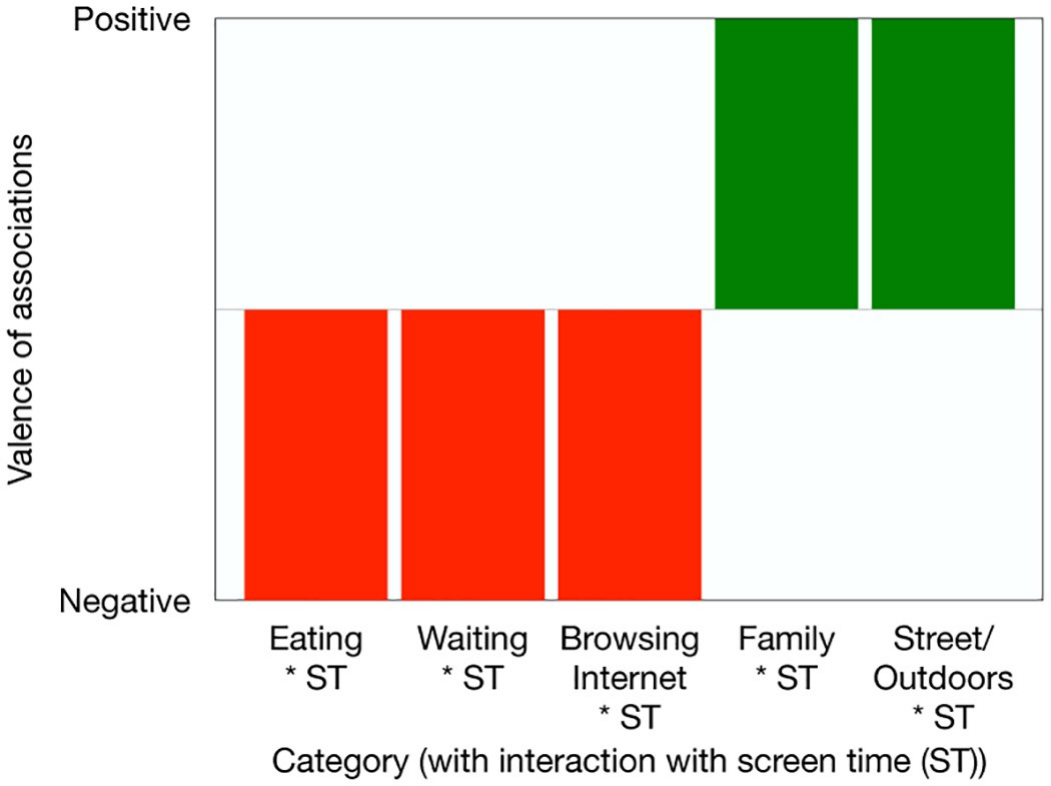
Valence of associations for interactions between context and screen time based on the results of the most complete model (using all contextual categories and screen time variables) from the regression analysis.

As we are mainly interested in the interactions between screen time and their context, with the regression models we reveal five interactions between context and screen time that are significantly associated with experienced happiness. Among the significant interactions, three are negative (eating, waiting and browsing Internet) and two are positive (family, street/outdoors), illustrated in Fig 5. We discuss these interactions and their interpretation in Section 7.3.

## 6 Qualitative survey

We conducted an online survey (*N* = 171) to better understand users’ perception towards the impact of smartphone usage on their well-being.

### 6.1 Participants and inclusion criteria

171 Participants were recruited using various sources: a crowd-sourcing website, personal contacts, and social media. The full survey can be found in the S2 Appendix. To evaluate the quality of data provided by each participant, we used three criteria: 1. The user took more than 5 minutes to answer the questionnaire, 2. The user owned a smartphone, 3. Their answer to a duplicated sanity check question. In particular, we presented the question: *’What is the highest degree or level of education you have completed or currently pursuing?’* twice in the survey; however, the order of options was jumbled in the second question. If the user selected different options for the two variations of the same question, they were discarded.

After filtering, we used the data from 143 participants (48 Female, 98 Males, 1 Other) for our analysis. Almost half of the participants were aged between 26–34 (N = 69), while others belonged to various age ranges, including 35–44 (N = 33), 18–25 (N = 20), 45–54 (N = 14), 55–64 (N = 5) and 65+ (N = 2). Additionally, they lived in different continents around the world including North America/Central America (N = 55), Asia (N = 53), Europe (N = 33), Africa (N = 1) and South America (N = 1).

### 6.2 Perceived impact of phone use

First, participants were asked to rate from 1–7 (1 being strongly negative, 4 neutral, and 7 being strongly positive) if they believed that using a smartphone had a negative or a positive impact on their well-being. Additionally, they were also asked to select from 1–7 (1 being strongly disagree) if they wanted to reduce their phone usage. On average, we observed that people believe that using smartphones has a slight positive impact on their well-being (*μ* = 4.8, *σ* = 1.35); however, they also slightly agree that would like to decrease their phone usage (*μ* = 4.37, *σ* = 1.60). This re-iterates that it is important to explore those situations in which phone usage is detrimental to well-being.

**Fig 6:**
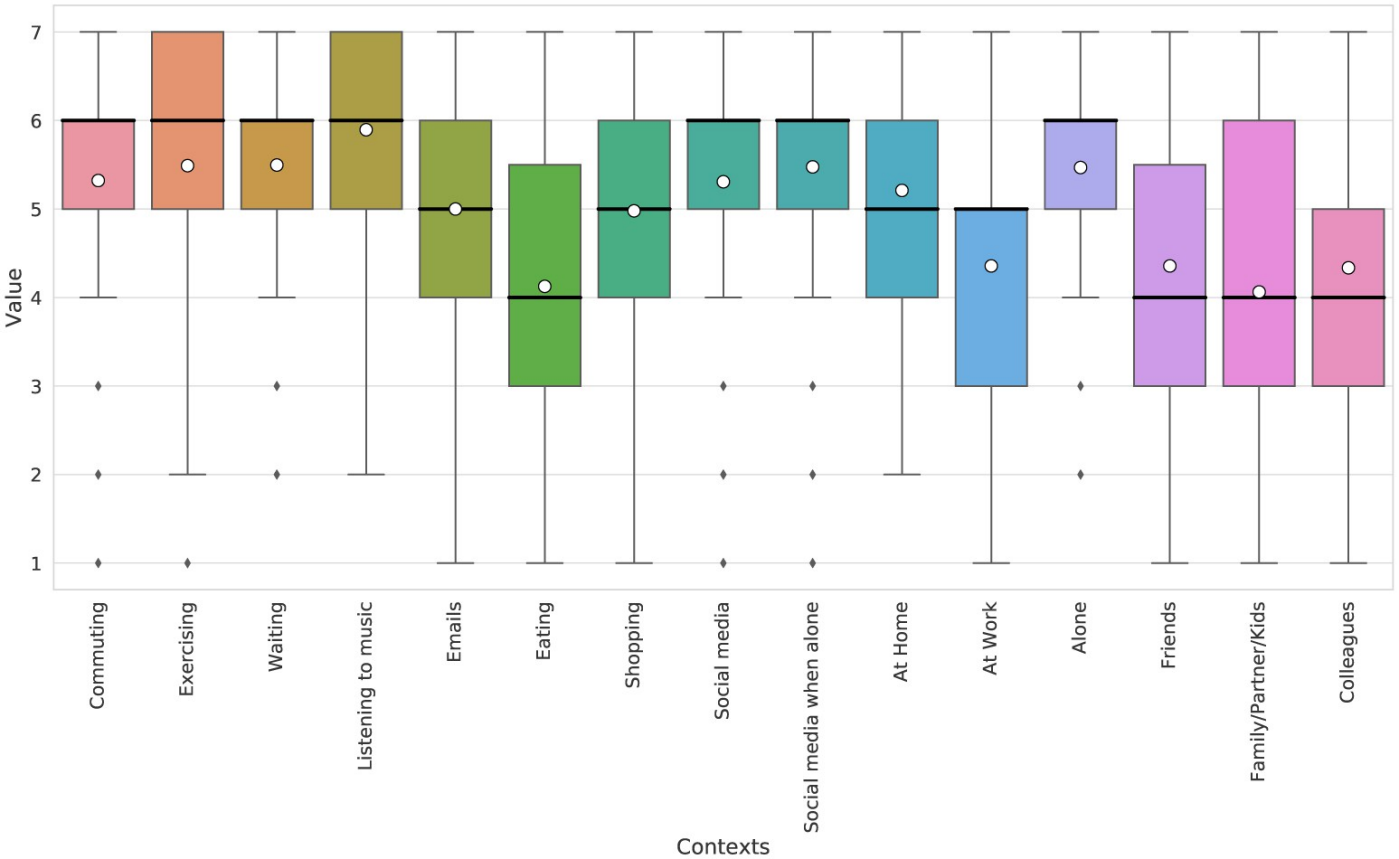
Box-plot of reported well-being ratings when smartphones are used in different contexts. In each box, the white circle represents the mean value, while the thick black line represents the median value of the reported well-being rating for that category.

Next, participants were presented with different contexts and asked if smartphone usage during these instances positively or negatively impacted their well-being. We selected the contexts that matched the most common activities, locations and people that were obtained from the self-reports in the quantitative study. Participants were asked to rate their well-being between 1–7 (1 being very negative) when using their phone during those contexts. A box-plot of these ratings, grouped per context is presented in Fig 6. We observed that people believe that their well-being is impacted more negatively when using smartphones during eating and when they are with company (friends, family, partners, kids and colleagues), compared to other circumstances. Moreover we observe a variation in the median and mean ratings across contexts, reinforcing the key finding from the quantitative study, i.e., that context impacts the perceived effect that smartphone usage has on peoples’ subjective well-being. Some additional observations from this qualitative analysis include:

1. Most people find their phone to be a useful resource, as long as it does not interfere with their ongoing activity (83.9% voted positively, 10.5% voted neutrally and 6.3% voted negatively)
2. Most people think that their phones help them feel connected and better, when they are alone (81.2% voted positively, 13.9% voted neutrally and 4.9% voted negatively)
3. Most people think that they spend the majority of their time on their phones using social media (74.1% voted positively, 7.7% voted neutrally and 18.2% voted negatively)
4. Most people use their phones to take a break from work (66.43%) rather than for working (18.18%), while the remaining use their phones for both.

### 6.3 Testimonials

In addition to asking users to rate their well-being during different contexts, we had two free-text questions to understand if there are certain experiences in participants’ everyday life that improve or worsen their well-being. These questions were: **(Q1)** *‘Are there certain experiences in your everyday life when using your smartphone improves your mood and makes you happy?’* and **(Q2)** *‘Are there certain experiences in your everyday life when using your smartphone worsens your mood and makes you unhappy?’* Most users answered with short texts that were less than 20 words in length. For these qualitative responses, we performed a simple clustering exercise to explore if their answers could be grouped into overarching themes or observations. The following observations emerged from their answers:

1. Smartphones improve well-being when they are used for their original intended purpose—talking and texting with friends and family. Answers to **Q1** included: “*When I talk or text with a friend*.”, “*…talking to friends through social media like Facebook and WhatsApp*…”, “*Using my smartphone makes me happy when I communicate with my friends or family*.”, and “*Talking to my friends when I’m feeling lonely helps a lot. Especially in these tough times*.”. Interestingly, many users specifically mention video calling—“*Video chatting with my family members decreases my work pressure*”, “*Making a video call with my wife while away from home improves my mood*.” and “*Video calling friends/family while doing housework*.”.
2. Listening to music with smartphones improves well-being: Numerous participants reported that listening to music or podcasts improves their well-being. Some answers to **Q1** related to music included: “*Listening to music and/or podcasts on my phone when I do exercise, sunbathe, etc. makes me feel good*.”, “*Listening to music when I’m on the street or at home doing stuff around the house and want to block any surrounding sounds*.”, “*Being able to listen to music while on public transportation to block out noise improves my mood*.” and “*When I’m prepping dinner, or getting ready to do a really stressful workload I’ll turn on apple music playlist on my iPhone/smartphone*”
3. Using smartphones to help with meditation, learning and exercise improves well-being: When smartphones are used as enablers to do these activities, users reported that it improves their well-being. Answers to **Q1** included: “*Listening to meditation, using it for exercise videos, using it to learn (i.e. a language), speaking to family and friends*”, “*I have several spiritual apps that send me notifications with positive messages throughout the day. They bring me good feelings and inspirations*.”, “*…Tracking my walking and cycling activities via GPS is also a great motivator (and the mapping is great for route finding)*”. Some users also reported that listening to music on their smartphones encourages them to exercise. Answers included: “*Listening to a favorite play list when going for a morning exercise has always made my mood awesome*.” and “*…I also listen to a lot of music, which encourages me to exercise and be active*.”.
4. Social media usage both improves and worsens well-being. Answers to **Q1** included: “*I usually use the mobile phone to see what’s coming up on social media. Although I am lonely when used, a feeling of being with others is prevalent*.”, “*Scrolling on social media improves my mood when I’m bored*”, “*When I have some alone time and I go on my phone to browse news and social media it improves my mood*.” and “*…usage of social media on my smartphone most especially twitter has always done great wonder to my mood*.”. However, answers to **Q2** included: “*…Using Instagram also worsens my mood, I can’t help and use it but it makes you compare your life with others’ and that may not always be so positive*.”, “*When I scroll through Facebook for 30 minutes, and realize I’ve just wasted 30 minutes*” and “*When I see something on social media that I don’t like too much and it affects me*.”.
5. Users reported that playing video games both improved and worsened their well-being. **Q1** answers included: *I play video games in my smartphone often and it helps me to be in a happy mood*. and “*Playing video games on my smartphone when I am less busy improves my mood*..”. **Q2** answers included: “*Sometimes by playing the games make me mood upset*.”, “*Playing longtime games makes me stressful*” and “*playing games*”.
6. Many users reported that using phones when you’re physically with friends and family worsens their well-being. Answers to **Q2** included: “*Using phone in the evenings when my husband has finished work. We both use our phones so don’t talk. Also—WhatsApp messages can be so distracting. There is so much pressure to respond to all the messages you receive from various platforms*”, “*…Using it while out with friends, as then we won’t have really meaningful conversations*.”, “*When my wife used her mobile phone and she doesn’t respond to me. That time I get irritated*.” and “*when I have family time, and a really urgent text comes through on my iPhone, family members get upset with me because my time is not focused on them, and then I become upset with myself and my phone usage*.”.
7. Users reported worse well-being when smartphones enable work life to invade personal and social life. **Q2** answers included: “*Non-stop messages and notifications from work chats and programs, work almost in each program we use (WhatsApp, Telegram, Facebook, Instagram, Notion, so on) -> no personal space and no silence. Even on holidays you are online and reachable. No respect to personal time (even at night is fine to write)*”, “*Using my phone to check work emails worsens my mood because my partner gets upset, but I can’t avoid it*.” and “*When I use it for work and it sometimes makes me feel as if I’m always on*…”.
8. Users also don’t like to read bad news on their mobile devices, indicated by these **Q2** answers: “*Reading news articles on my phone about sad things going on in the world puts me in a bad mood*”, “*Seeing bad news*”, “*when there are same stories like Facebook news and other things nothing to cheer you up and you miss something real, that time it worsen the experience*.”, “*some times WhatsApp messages from groups about some political nature make me so uncomfortable, because i know the reality of that messages and they are created only from the frustration or agony against the opposite groups*”.

## 7 Discussion

### 7.1 Effect size of screen time on subjective well-being

Both the correlation and regression analyses in this study suggest a weak association between screen time and subjective well-being. This is inline with recent reports, such as [41, 42] which argued that there is a lack of strong evidence on the detrimental effects of smartphones use on our mental health [41, 42] and that previous studies and systematic reviews overestimated the negative associations [4]. Importantly, our study highlighted the role of daily contexts in the associations between the phone use and subjective well-being. In other words, previous research may be wrong in analysing the two in vacuum—in reality, it appears that it is about the interplay between screen time and contexts that drive positive and negative associations with well-being.

### 7.2 Accumulated effect is negative, yet not all screen time is deleterious

Consistent with the previous literature, we found that the overall phone use association with subjective well-being is a negative one, regardless of context (see Fig 4, and regression coefficient in (1) in Table 3). However, the effect disappeared in the regression analysis when considering different contextual information.

### 7.3 Interactions between screen time and context: Regression analysis

The regression results show that in the configurations where contextual features were added, the screen time features lost their significance. We revealed five interactions that remained significant when adding both context and screen time features. The first is that while eating has a significantly positive correlation (*p* < 0.001) with experienced happiness, the association of its interaction with screen time and happiness is significantly negative (*p* < 0.05). This indicates that eating is positively associated with experienced happiness, but the use of the phone while eating has a negative component. Moreover, we’ve found that using the screen time in the presence of company produces a weak positive association with experienced happiness. We hypothesised that this may happen in the presence of people with whom the user is comfortable with, such as partner and family (e.g., leisure time before bed time). We encourage further research to dive and dissect these results, as it would be interesting to discover for instance in which cultures this happens, as well as in the presence of which company (since partner may be labelled as family as well).

While the previous results are not unexpected, interestingly, we hypothesised that while waiting, the user may find screen time as a positive activity (e.g., for communicating with others, for social media, for productive activities). Surprisingly, we’ve found a strong evidence (*p* < 0.001) that the using the phone during waiting is negatively associated with experienced happiness (both in correlation and regression analyses). One possible explanation for this is that not all idle time should be filled with screen time, and one should preserve this time for reflection. Another possible interpretation of this result is that when we have a lower experienced happiness while waiting, we use the phone more.

Finally, we discovered the the use of phone in the outdoors or on the street was weakly but significantly positively associated with experienced happiness. This may be due to facilitator apps (e.g., offering directions, reviews of nearby places, etc.), yet we hypothesised that using the phone in outdoor activities would have the opposite effect. We encourage further research in this line to discover which outdoor activities were positively associated with experienced happiness.

### 7.4 Perceived impact from qualitative study against observed associations from quantitative study

Our main finding is that indeed people report that context plays a big role into whether they perceive the impact of the phone use on well-being as positive or negative. Moreover, while we did not explore causal links in the quantitative study analysis, the qualitative study allowed us to explore suggestions of causal links. When diving deeper into people’s testimonials, it becomes evident that the majority of statements include at least one context that influences the association between phone use and well-being. For instance, users reported that when being on holidays or with family, receiving a text on their smartphone related to work causes a decrease in their well-being, shedding a light on the importance of considering the context when evaluating the impact of phone usage on well-being. Moreover, we observe that certain smartphone uses are perceived to be both positive and negative, such as using the phone for social media alone, versus scrolling for a long time, which are observable passively only when combined with available contextual information, in this case activity and company, and duration.

We further compared the perceived effects of smartphone use on well-being from the qualitative survey responses with the findings from the quantitative study. Some differences and similarities arise. For instance, we observed that people perceive that their well-being is negatively affected when they are with company (friends, family, partners, kids and colleagues), while it is positively impacted when they listen to music and exercise. While the quantitative study indicates that well-being does improve when people listen to music or exercise, it also shows that there is a positive association between well-being and phone use when people report being in the company of family or their partner. Moreover, qualitative data does give us a layer of depth that is difficult to obtain from quantitative data. As an example, people provided very detailed responses under which circumstances certain smartphone uses were positive or negative. Using the free-text entries from our qualitative data, we noted that playing video games or using social media both improves and worsens mood, and people were very descriptive of the contexts when this happens. Bringing together both subjective views and objective measurements allowed us to further highlight the importance of considering context when evaluating smartphone usage impact on well-being.

### 7.5 Differences in associations between the two considered dimensions of well-being, happiness and worthwhileness

We observed a Spearman correlation coefficient of 0.76 (*p* < 0.001) between happiness and worthwhileness. The high correlation between the two metrics suggests that the two dimensions of well-being do not differ largely from each other for the sample population under observation. Indeed, we did not find a single difference in direction of association between screen time and one dimension of well-being against the other, i.e. there are no associations that are positive with happiness and negative with worthwhileness or the opposite. In general, the insights observed for happiness and worthwhileness are in line and coincide when considering the direction of association with screen time. Further research could dive deeper to explore whether there are any differences between the well-being dimensions when considering individual differences in well-being.

### 7.6 Duration against relative percentage in various contexts

In particular, we are interested in answering whether there are any differences in associations when looking at the total duration of screen time during an EMA episode compared to the percentage of screen time during an episode. We observed a Pearson correlation coefficient of 0.51 (*p* < 0.001) between percentage duration and total duration of screen time. In general, the insights observed from the total duration of screen time and percentage duration of screen time are in line and coincide when considering the direction of association. The strength of the association and the significance may vary across the two depending on the context (e.g., Extroverts in Fig 4).

### 7.7 Implications

The main contribution of our study stems from demonstrating how a set of factors influence the complex relationship between phone use and well-being beyond the mere screen time as per the previous literature. By combining qualitative and quantitative analysis, our study contributes to the HCI community by shedding light on the interrogations of recent reviews as well as on the conclusions in the popular press typically categorical about either the positive or negative impact of screen time (mostly the latter, which makes the headlines). This investigation’s results show how context plays a big role into whether the associations with reported well-being are positive or negative.

There is much potential for further innovation of designing for well-being as well as in the space of digital phenotyping and individual tracking apps that give insights into everyday screen time and device use. Church et al. [60] say that improved user interfaces and user experience is likely to result in better data collection in device tracking studies. Given that most collection apps nowadays rely on users opening the app and engaging with it to avoid missing data, especially for Android phones, displaying insights into users’ contextualized screen time and well-being could engage users towards a more accurate passive data collection, as it would offer information back to the user, complementary to collection only. Moreover, people report that context plays a big role on whether they perceived phone use as negative or positive for their well-being. Thus, we suggest that HCI’s interest in individual data collection tracking apps should not be limited only by passive data collection, but should engage users and provide insights such that they make sense of their screen time beyond device and application usage, to better reflect on how to organize their everyday lives.

Moreover, designing mobile phones to raise awareness to users of how the context of phone use plays a role into their individual well-being could potentially lead to an improvement in their well-being. We envisage a future application, where depending on the valence of the association, the user would be prompted to reflect on whether the phone use in that (passively detected or reported) context is necessary at that time and requires their full attention. Such design maintains users’ control on their phone use, but at the same time prompts the user on reflecting whether the potential negative impact on their well-being can be avoided. Such prompts could be particularly targeting those situational contexts that were shown to have a negative association with well-being in this study.

Finally, further innovation for designing virtual interactions through mobile phones during various contextual situations could be explored, such as leveraging positive or negative rewards depending on the context. One such example is given in [61] where the context at play is having a meal. The authors suggest an interactive game between the individuals at the table sharing a meal where they are given a positive reward when not engaging with the mobile phone, (e.g., an ice cream sundae badge for not going online or using social media during a meal). Similarly, the insights from this study can be used to guide the design of such interactive games where rewards are given only in situational contexts that have been shown to play a significant role in the association of screen time with well-being, and we suggest that the rewards are tuned based on the valence and significance of the association.

### 7.8 Limitations

One limitation of our study is that we rely on self-reported well-being measures. Recent studies exploit latest developments in automated emotions detection, such as the study by Sarsenbayeva et al. [40] which uses the Affectiva Emotion SDK [62] to automatically detect emotions from facial expressions and map them to passive smartphone sensing measurements. The study also collects self-reports to triangulate against and check the accuracy of the Affectiva data. We envisage a future study where the collection of target well-being and emotional states can be done continuously and passively in time as the users use their smartphone to avoid relying on self-reports for the target variables completely.

Another limitation of this study is the fact that the screen time is measured only during the duration reported in the EMAs collected in order to obtain a mapping between context and smartphone use. Therefore, the findings can be affected by users’ memory, as the duration of an activity may be reported only approximately. This mainly affects the raw total duration screen time measurement, where we measure the amount of time the user spent on the phone during the context reported. We partly addressed this limitation by computing the percentage screen time measurement, as it is relative to the duration reported in the EMA. A more advanced potential solution to this problem is to explore acquiring contextual information in a passive manner as well.

An additional limitation is that our study was not designed to explore causal effects on the EMA’s. To study the causal links, the study would have to have captured experienced happiness before and while performing the activities reported in the EMA’s to see how experienced happiness changes according to screen time usage within each context. Such study would be very difficult to put in practice in the wild as it requires knowing when the participant is going to start an activity and prompting the participant for self-reports while the activity is ongoing. For that reason, the analyses performed in this paper present associations between experienced happiness and screen time together with context, and we refrain from making firm claims on the causal effects of screen time on experienced happiness.

Finally, we explored users’ perception of screen time impact in various contexts using a different sample population, mainly due to the initial study being ran for a wider list of hypotheses and completed more than a year ago. We were still interested in how users perceive smartphone use in different contexts and therefore ran a separate qualitative survey to enrich this study from this dimension. Another limitation of this survey is that the recruitment was done through social media and similar means, and thus the results may suffer from this selection bias.

### 7.9 Future research directions

In our study participants belonged to a plethora of countries, both in the quantitative and qualitative studies, as an extra consideration was given to ensure that a diverse cross-country sample is collected. Yet our study was not designed to evaluate the cross-cultural aspect of the phone use relationship with well-being. We encourage future studies with a balanced sample across more cultures covering different continents to explore the role of culture in the relationship between screen time and well-being.

Moreover, even though we attempted to remove the subjectivity by collecting passively the screen time, we still relied on active data for reporting activities duration and self-reported well-being measures. We encourage a future study to look at leveraging latest methods in emotional states detection and context (activities, companies and location are a few of the contexts that have been explored and promising methods emerged for their passive inference in [63–67], to name a few).

Subjective reports are still important as we can see from the testimonials from the qualitative study, which reveal that even the same context can trigger different responses and lead to both worsening or improving a user’s well-being based on other factors, such as how the user is currently feeling. Reports that social media both improves (when feeling lonely or bored) and worsens (when wasting time) well-being highlight how useful it is to consider a persons well-being in the first place, when diving into the valence of the associations between well-being and screen time.

Finally, we encourage work in this area to dive deeper into deviant individuals and deviant screen time use associations with well-being. In particular, it would be interesting to discover whether happy or unhappy individuals (for instance as classified based on their individual SWB set-points) rate differently their SWB in different phone use contexts. Moreover, we encourage future work to explore the “threshold” for problematic screen time use, and the associations between SWB and screen time for deviant individuals (for instance as defined based on their screen time use). Lastly, we encourage future work to explore the associations between SWB and deviant screen time uses (for instance as defined per individual based on screen time distribution) in different contexts.

## 8 Conclusion

Smartphones have become a central part of our daily lives, regardless of the controversy around its effects on our well-being. Prior studies have argued in plentiful numbers that using smartphones is associated with poorer well-being [17, 24] and mental health [2, 19]. Yet recent studies [4, 43] challenged these claims and argued that there is very little evidence on the negative effects of smartphones on our well-being and that previous studies have overestimated the effects. Diving deeper into the associations of smartphone use with well-being, this study reveals other important factors to consider, such as surrounding context (such as activity, location, company) and personal characteristics. We present detailed results under which factors phone use is associated with a positive increase in well-being or a negative decrease in well-being, leveraging passive smartphone sensing data coupled with active user reports on their context and well-being. Finally, we compare these results with the perceived effects of smartphone use on well-being, extracted from a subsequent qualitative study. Our results emphasize that context plays a big role into whether the associations with subjective well-being are positive or negative. Moreover, positive associations between screen time and well-being were discovered, along with negative ones, however all in all the strength of these associations was weak. Our paper highlights the complexity of this problem and reveals an obscured hidden part of the screen time iceberg.

## Supporting information

S1 Fig Box plot of subjective well-being in different contexts per activity. (PDF)

S2 Fig Box plot of subjective well-being in different contexts per company. (PDF)

S3 Fig Box plot of subjective well-being in different contexts per location. (PDF)

S1 Appendix Subjective well-being in different contexts. (PDF)

S2 Appendix Survey used for qualitative study. (PDF)
