# Supplementary figures and images for: Two edges of the screen: Unpacking positive and negative associations between phone use in everyday contexts and subjective well-being
Source: PLoS One. 2023 Apr 26;18(4):e0284104. doi: 10.1371/journal.pone.0284104 (PMC10132652; doi:10.1371/journal.pone.0284104)

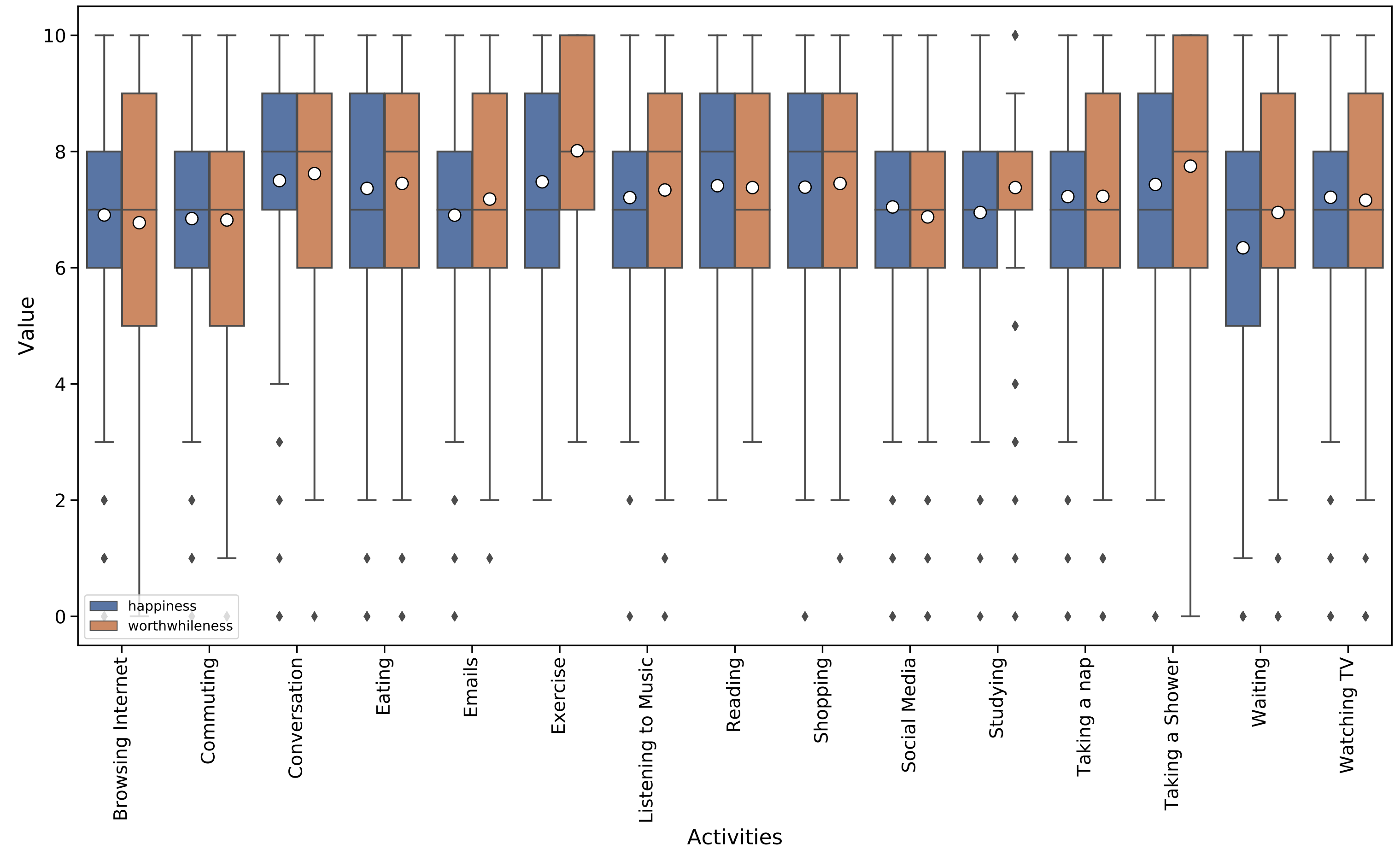

Supplement: S1 Fig — (PDF) [file pone.0284104.s001.pdf]

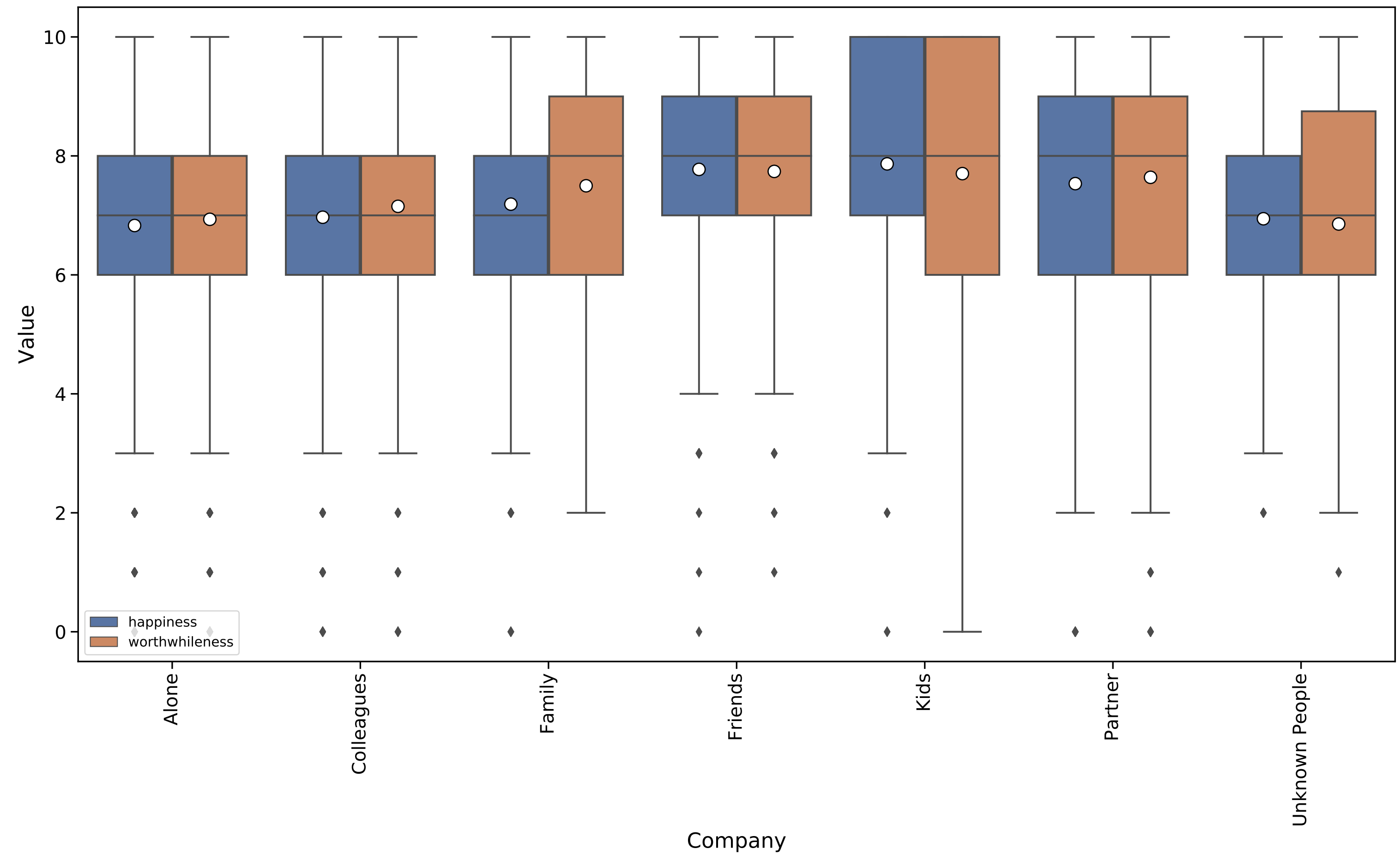

Supplement: S2 Fig — (PDF) [file pone.0284104.s002.pdf]

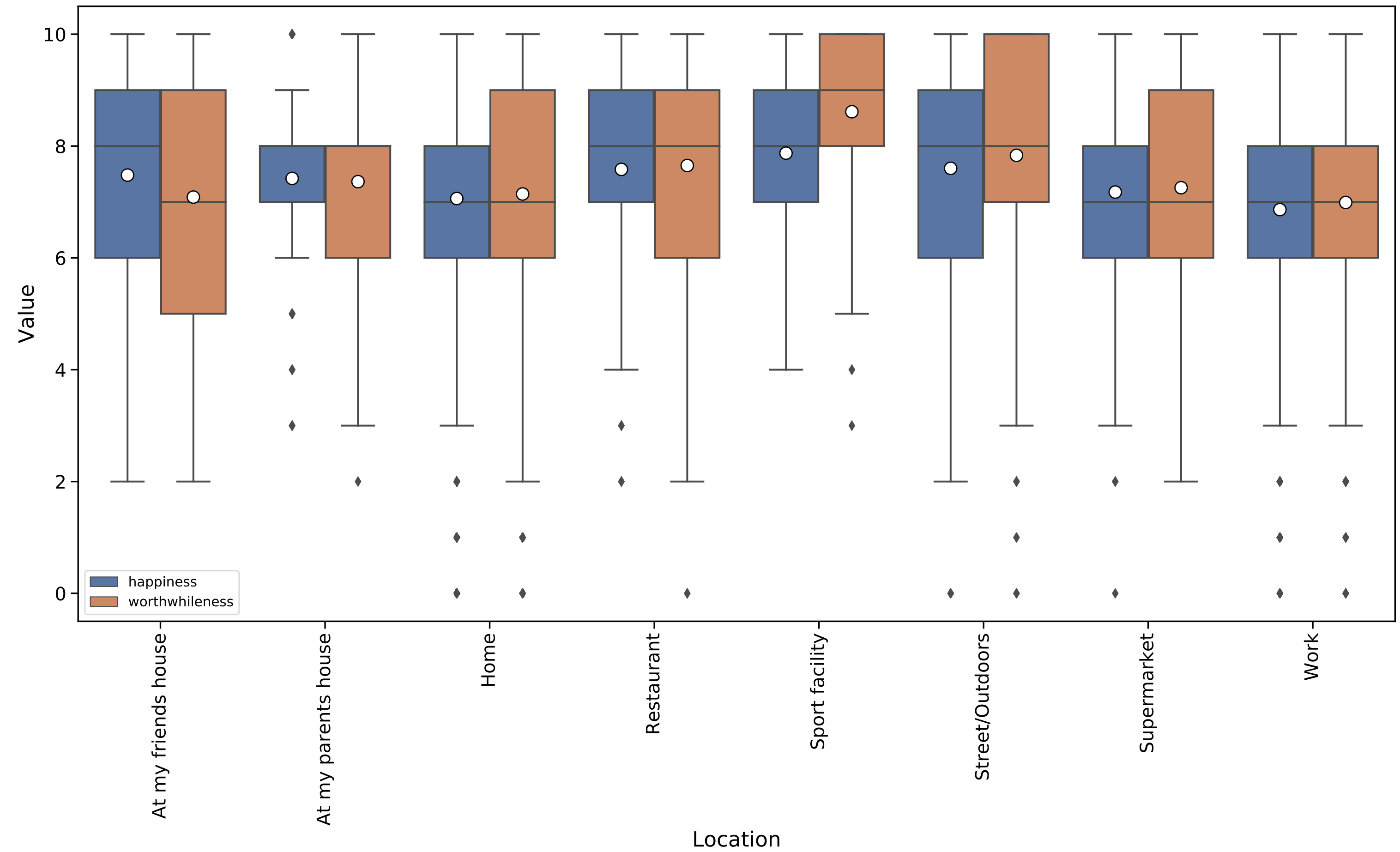

Supplement: S3 Fig — (PDF) [file pone.0284104.s003.pdf]
