# Supplementary material for: Two edges of the screen: Unpacking positive and negative associations between phone use in everyday contexts and subjective well-being
Source: PLoS One. 2023 Apr 26;18(4):e0284104. doi: 10.1371/journal.pone.0284104 (PMC10132652; doi:10.1371/journal.pone.0284104)
Supplement: S1 Appendix — (PDF) [file pone.0284104.s004.pdf]

Two Edges of the Screen: Unpacking Positive and Negative Associations between Phone Use in Everyday Contexts and Subjective Well-being

Supplementary Material

Appendix 2: Survey used for qualitative study

Screen 1: Welcome

Screen 2: Thank you for being a part of this research study.

The main goal of our study is to understand how using smartphones influences your mood and happiness.

It is expected that you will not spend more than 30 minutes to complete this questionnaire, and we believe that reflecting on the impact of phones on your daily life will be insightful to you. Your participation in this study is completely anonymous.

It is important that you provide honest answers to all the questions, as your responses will be used to improve the mental health of other people.

By clicking ’Continue’, you agree to answer the questions in this study honestly.

Screen 3: We’d like to know which demographic do you belong to

Q: What is your gender?
Options: Female
Male
Other

Q: What is your age?
Options: <18
18-25
26-34
35-44
45-54
55-64
65+

Q: What is the highest degree or level of education you have completed or currently pursuing?
Options: No education
Primary School
Secondary School

Bachelor's Degree
Master's Degree
Ph.D. or higher
Trade School
Other

Q: Where do you currently live?
Options:North America/Central America
South America
Europe
Africa
Asia
Australia
Caribbean Islands
Pacific Islands
Other

Screen 4: Now, we would like to understand about your experiences with a smartphone

Q: Do you own a smartphone?
Options: Yes
No

Q: On average, how long do you use your smartphone in a day?
Options: <30 mins
30 mins - 1 hour
1-2 hours
2-3 hours
3-4 hours
4-5 hours
5-6 hours
6-7 hours
7-8 hours
>8 hours

Q: Generally speaking, would you like to decrease your phone usage?
Options: Likert scale - 1 (Strongly Disagree) to 7 (Strongly Agree)

Q: Overall, do you think that the smartphone usage has a positive or a negative impact on your mood?
Options: Likert scale - 1 (Very Negative) to 7 (Very Positive)

Screen 5: Smartphones have become a prominent part of our daily lives. We use them often, even while doing another activity - for instance, while eating, waiting in a supermarket, commuting, etc.

Using your smartphone while doing something else can make you feel better (e.g., if you are in a very boring activity), or it can make you feel worse (e.g., if by using your phone you are missing an interesting conversation) or it can be neutral experience (e.g., scrolling your Twitter news feed)

Q: Are there certain experiences in your everyday life when using your smartphone improves your mood makes you happy?
Be as specific as possible. e.g., Listening to music on my phone when I walk from work to home improves my mood
-Free-text response-

Q: Are there certain experiences in your everyday life when using your smartphone worsens your mood makes you unhappy?
Be as specific as possible. e.g., Using my phone when I'm on a date with my partner leads to fights between us and this makes me upset.
-Free-text response-

Screen 6: Think carefully about how using your phone during the following activities impacts your mood during or shortly after that activity.
If it helps, try to remember the last time you were doing these activities and using the phone; even a brief phone use counts.
Indicate the impact corresponding to each activity

Options for each: Likert scale - 1 (Very Negative) to 7 (Very Positive)

Commuting
Doing productive work (e.g., working, studying, or similar)
Exercising (e.g., jogging, gym, etc.)
Waiting (e.g., in a supermarket, in a bank, etc.)
Listening to music (with or without your phone)
While sending emails
Eating
Shopping
When you use your mobile phone for social media
When you are alone, and use your mobile phone for social media
When you are at home (whichever activity you are doing)
When you are at your workplace (whichever activity you are doing)
When you are alone (whichever activity you are doing)
When you are with your friends (whichever activity you are doing)
When you are with your family/partner/kids (whichever activity you are doing)
When you are with your colleagues or classmates (whichever activity you are doing)

Q: What time of the day does using your smartphone improve your mood?
Options: Early Morning (6am-9am)
Late Morning (9am-12pm)
Afternoon (12pm-6pm)
Early Evening (6pm-9pm)

Late Evening (9pm-12am)
Night (12am-6am)
None of the above

Q: What kind of apps do you use on your smartphone phone when you want to improve your mood and to feel better?
Music/Podcasts/Audiobooks
Video streaming
Health
Messaging, including WhatsApp, Telegram etc.
Sports/Exercise
Social Media
News
Emails
Shopping
Web browser
Banking
Games
Calls
Other

Q: On an average day, what kind of smartphone apps do you regularly use?
Music/Podcasts/Audiobooks
Video streaming
Health
Messaging, including WhatsApp, Telegram etc.
Sports/Exercise
Social Media
News
Emails
Shopping
Web browser
Banking
Games
Calls
Other

Q: Is most of the time that you spend on your smartphone spent on social media websites and apps like Facebook, Twitter, Instagram, Snapchat, TikTok etc.?
Options: Likert scale - 1 (Strongly Disagree) to 7 (Strongly Agree)

Q: Do you use your mobile phone during working hours more for work or to take a break from work?
Options: Work
Take a break from work
Both

Screen 7: Please select a number next to each question to answer each question.

Options for each: Likert scale - 1 (Very Low) to 10 (Very High)

Q: Overall, how satisfied are you with your life nowadays? Q: Overall, to what extent do you feel the things you do in your life are worthwhile? Q: Overall, how happy do you feel in general? Q: Overall, how anxious do you feel in general?

Screen 8: Q: How do you associate with the following statements?

Options for each: Likert scale - 1 (Strongly Disagree) to 7 (Strongly Agree)

I have used my mobile phone to make myself feel better when I was feeling down.
When out of range for some time, I become preoccupied with the thought of missing a call.
If I don't have a mobile phone, my friends would find it hard to get in touch with me.
I feel anxious if I have not checked for messages or switched on my mobile phone for some time.
My friends and family complain about my use of the mobile phone.
I find myself engaged on the mobile phone for longer periods of time than intended.
I am often late for appointments because I'm engaged on the mobile phone when I shouldn't be.
I find it difficult to switch off my mobile phone.
I have been told that I spend too much time on my mobile phone.
I have received mobile phone bills I could not afford to pay.
As long as the phone does not interfere with my ongoing activity, I find it a very useful resource (e.g., using a map app while commuting, listening to music while exercising, etc.)
When I'm alone, the phone makes me feel connected and better.

Q: What is the highest degree or level of education you have completed or currently pursuing?
Options: No education
Primary School
Secondary School
Bachelor's Degree
Master's Degree
Ph.D. or higher
Trade School
Other

Screen 8: You're almost done! Here are a number of statements that may or may not apply to you. Please select a number next to each statement to indicate the extent to which you agree or disagree with that statement.

Options for each: Likert scale - 1 (Strongly Disagree) to 7 (Strongly Agree)

I am the life of the party.
I sympathize with others' feelings
I get chores done right away.
I have frequent mood swings.
I have a vivid imagination.

I don’t talk a lot.
I am not interested in other people’s problems.
I often forget to put things back in their proper place.
I am relaxed most of the time.
I am not interested in abstract ideas.
I talk to a lot of different people at parties.
I feel others’ emotions.
I like order.
I get upset easily.
I have difficulty understanding abstract ideas.
I keep in the background. ll
I am not really interested in others.
I make a mess of things.
I seldom feel blue.
I do not have a good imagination.

Screen 9: Thank you for completing the survey. We appreciate your time.
